# Untersuchung von SARS-CoV-2-Ausbrüchen in Deutschland durch Feldteams des Robert Koch-Instituts, Februar–Oktober 2020

**DOI:** 10.1007/s00103-021-03296-y

**Published:** 2021-03-17

**Authors:** Katharina Alpers, Sebastian Haller, Udo Buchholz, RKI Feldteam, Muna Abu Sin, Maria an der Heiden, Jennifer Bender, Sonia Boender, Michael Brandl, Tim Eckmanns, Christina Frank, Ariane Halm, Uwe Koppe, Raskit Lachmann, Marina M. Lewandowsky, Matthäus Lottes, Inessa Markus, Dorle Matysiak-Klose, Kai Michaelis, Felix Moek, Nadine Muller, Kirsten Pörtner, Sybille Rehmet, Andreas Reich, Felix Reichert, Neil Saad, Navina Sarma, Kathrin Schaten, Timm Schneider, Madlen Schranz, Regina Selb, Gyde Steffen, Anna Stoliaroff-Pépin, Sabine Vygen-Bonnet, Jan Walter, Nadine Zeitlmann, Ruth Zimmermann

**Affiliations:** Abteilung für Infektionsepidemiologie, Robert Koch Institut, Seestr. 10, Berlin, Deutschland

**Keywords:** COVID-19, Ausbruchsuntersuchung, Ausbruchsmanagement, Nosokomiale Ausbrüche, Feldepidemiologie-Trainingsprogramm, COVID-19, Outbreak investigation, Outbreak management, Healthcare-associated outbreak, Field epidemiology training program

## Abstract

Das Robert Koch-Institut (RKI) bietet den Gesundheitsämtern in Deutschland bei Ausbrüchen von Infektionserkrankungen Beratung und praktische Unterstützung vor Ort an. Die Feldeinsätze werden von speziell geschultem Personal durchgeführt. Auch im Rahmen der COVID-19-Pandemie leistet das RKI regelmäßig diese Form der Amtshilfe in unterschiedlichen Settings. Dabei handelt es sich beispielsweise um Ausbrüche in Wohngebäuden, Arztpraxen, Alten- und Pflegeheimen, Kliniken, Erstaufnahmeeinrichtungen für Asylsuchende, aber auch in einem Nachtclub oder auf einem Kreuzfahrtschiff.

Der vorliegende Beitrag berichtet exemplarisch von Feldeinsätzen, die im Zeitraum Februar bis Oktober 2020 im Rahmen der COVID-19-Pandemie stattgefunden haben. Die daraus gewonnenen Erkenntnisse tragen dazu bei, das Wissen zu SARS-CoV‑2 zu erweitern, z. B. zur Übertragung und Ausbreitung des Erregers, RKI-Empfehlungen zu formulieren oder zu untermauern und das Management komplexer Situationen zu unterstützen. Die Praxisbeispiele zeigen, wie vielfältig die RKI-Teams nicht nur vor Ort unterstützen, sondern auch die epidemiologische Evidenzbasis bereichern.

Im September 2020 wurde im RKI die „Kontaktstelle für den öffentlichen Gesundheitsdienst (ÖGD)“ eingerichtet, die unter anderem die Amtshilfe durch Feldeinsätze koordiniert und erweiterte Beratungsmöglichkeiten bietet. Damit der ÖGD langfristig noch besser auf Ausbrüche von Infektionserregern reagieren kann, soll das interdisziplinäre Trainingsangebot intensiviert werden.

## Abstract (en)

The Robert Koch Institute (RKI) offers advice and assistance to local public health agencies on the investigation and management of outbreaks of infectious diseases. Specially trained experts conduct field investigations. During the COVID-19 pandemic, RKI experts participated in the investigation of outbreaks in residential buildings, physicians’ offices, nursing homes, hospitals, and asylum seekers’ facilities as well as at a night club and on a cruise ship.

This report describes some of the field investigations conducted in Germany during the COVID-19 pandemic between February and October 2020. The investigations provided information on the properties of SARS-CoV‑2 and its transmission as a basis for the recommendations on suitable prevention measures. The practical examples demonstrate the variety of support given as well as the opportunities to gather epidemiological evidence.

In September 2020, the RKI established a new unit called the “Focal Point for the Public Health Service,” which now coordinates and is expanding field support. In order to further support the public health system to improve its capability to react to and investigate outbreaks of infectious diseases, interdisciplinary training shall be intensified.

## Einleitung

Im Dezember 2019 wurde der Ausbruch einer neuen Atemwegserkrankung in China bestätigt. Bereits am 30.01.2020 erklärte die Weltgesundheitsorganisation (WHO) den Ausbruch des Erregers „Schweres akutes Atemwegssyndrom-Coronavirus-Typ 2“ (severe acute respiratory syndrome coronavirus type 2 – SARS-CoV‑2) zur gesundheitlichen Notlage internationaler Tragweite (Public Health Emergency of International Concern – PHEIC; [1]). Gleichzeitig wurden auch in Deutschland die ersten Infektionen nachgewiesen.

Die Epidemiologie der Pandemie durch die Coronavirus-Krankheit-2019 (coronavirus disease 2019 – COVID-19) ist maßgeblich geprägt durch Fallhäufungen von SARS-CoV-2-Infizierten, die in verschiedenen Settings auftreten. Analysen dieser Ausbrüche sind eine Gelegenheit, das Infektionsgeschehen und den Erreger besser verstehen zu lernen. Die Suche nach Infektionsquellen durch die Gesundheitsämter kann hier wichtige Informationen liefern. Die Arbeit der Gesundheitsämter besteht darin, neben dem Management von Fällen und der Kontaktpersonennachverfolgung auch das wahrscheinliche Infektionsumfeld der Fälle zu ermitteln, um gezielte Maßnahmen ergreifen zu können. Beispielsweise können bei einer nachgewiesenen Ansteckung in einem Gruppensetting noch viele weitere Fälle unentdeckt sein. Die Informationen zum Infektionsumfeld von Ausbrüchen werden von den Gesundheitsämtern nach Infektionsschutzgesetz an die zuständigen Landesbehörden und das Robert Koch-Institut (RKI) übermittelt. Eine erste Analyse dieser Daten zeigt, dass von 202.225 bis zum 11.08.2020 übermittelten Fällen insgesamt 55.141 (27 %) mindestens einem Ausbruchsgeschehen zugeordnet werden konnten [2]. Die weitaus meisten Ausbrüche mit Angabe zum Infektionsumfeld fanden im familiären und häuslichen Umfeld statt, gefolgt von Ausbrüchen in Alten- und Pflegeheimen. Die größten Ausbrüche traten in Flüchtlings- und Asylbewerberheimen, in Alten- und Pflegeheimen sowie in Seniorentagesstätten auf.

Der rasche Anstieg von Fallzahlen, das Auftreten von Ausbrüchen sowie spezifische, lokale Probleme und komplexe Situationen können Gesundheitsämter vor außerordentliche Herausforderungen stellen. Obwohl die meisten Gesundheitsämter diese Herausforderungen innovativ, fachlich angemessen und unter immensem Einsatz bewältigen, gibt es auch immer wieder Situationen, in denen die zuständigen Landesbehörden oder das RKI um Amtshilfe gebeten werden (Infobox 1). Je nach Bedarf wird dann ein Feldteam aus 1–5 RKI-Mitarbeitenden zusammengestellt, das vor Ort Unterstützung leistet. Das entsandte RKI-Personal reiht sich immer in das entsprechende Ausbruchsteam vor Ort ein, und steht gleichzeitig mit weiterem erfahrenen Personal am RKI in Kontakt, welches zusätzlich beratend zur Verfügung steht. Eine zentrale Rolle in dieser interventionsepidemiologischen „Taskforce“ spielen die Teilnehmerinnen und Teilnehmer der Postgraduiertenausbildung für angewandte Epidemiologie (PAE), die als deutsches Feld-Epidemiologie-Trainings-Programm (FETP) am RKI angesiedelt ist (siehe Infobox 2). Zur Unterstützung der Gesundheitsämter vor Ort bei der Bewältigung der Kontaktpersonennachverfolgung wurden darüber hinaus sogenannte Containment Scouts (CS) eingeführt (siehe Infobox 3). Vor der Beantwortung evtl. sich ergebender wissenschaftlicher Fragestellungen steht die ganz praktische Unterstützung vor Ort im Vordergrund. Von Anfang Februar bis Mitte Oktober 2020 sind insgesamt 28 RKI-Teams Amtshilfeersuchen gefolgt, die RKI-Mitarbeitenden waren insgesamt über 330 Tage vor Ort.

### Infobox 1 Amtshilfe durch das Robert Koch-Institut (RKI)

- Gesundheitsämter können über die für den Infektionsschutz zuständigen Behörden der Bundesländer ein Amtshilfeersuchen an das RKI stellen.
- Primäres Ziel eines jeden RKI-Einsatzes ist die Unterstützung des lokalen Bedarfes. Dieser sollte möglichst genau gefasst und formuliert werden, damit effektiv und effizient reagiert werden kann. In einer Telefonkonferenz zwischen den beteiligten lokalen, Landes- und Bundesbehörden werden zu Beginn der Unterstützungsbedarf und die Zielstellungen formuliert. Folgende Fragen sollten beantwortet werden:
  1. Welche Expertise wird prinzipiell benötigt?
  2. Ist das RKI die am besten geeignete Institution für den entsprechenden Bedarf?
  3. Wie viele Personen werden benötigt, mit welchem fachlichen Hintergrund und für in etwa welche Dauer?
- Unterstützungsmöglichkeiten sind:
  - Telefonische Beratung zum Vorgehen/Management
  - Unterstützung bei Laboruntersuchungen
  - Vorübergehende Unterstützung der Gesundheitsämter bei der Infektionsquellensuche bzw. Kontaktpersonennachverfolgung
  - Unterstützung im Management der Gesamtsituation
  - Unterstützung in der Beantwortung spezifischer, lokaler Fragestellungen
- Je nach Bedarf werden von RKI-Seite für jede Situation etwa 1–5 Personen zu einem Feldteam zusammengestellt.
  Wissenschaftliche Mitarbeiterinnen und Mitarbeiter mit verschiedenen Erfahrungsstufen bzw. Ausbildungshintergrund (z. B. Epidemiologie, Mikrobiologie) werden dabei eingesetzt.

### Infobox 2 Feld-Epidemiologie-Trainings-Programme (FETP)

- Die Postgraduiertenausbildung für angewandte Epidemiologie (PAE) wurde 1996 als deutsches Feld-Epidemiologie-Trainings-Programm (FETP) am RKI eingerichtet.
- In enger Kooperation mit dem European Centre of Disease Prevention and Control (ECDC) und anderen nationalen Trainingsprogrammen lernen die Fellows, infektionsepidemiologische Methoden unter enger, fachkundiger Supervision auf aktuelle Public-Health-Fragestellungen anzuwenden.
- Die Fellows werden in systematischer Ausbruchsuntersuchung weitergebildet und sammeln Praxiserfahrung.
- Die FETP-Teilnehmer (sogenannte Fellows) in Deutschland sind am RKI für 2 Jahre angestellt und zum Teil auch in einigen für den Infektionsschutz zuständigen Behörden der Bundesländer angesiedelt [16].
- Die Fellows nehmen bei den Feldeinsätzen des Robert Koch-Instituts eine zentrale Rolle ein, indem sie Teil der interventionsepidemiologischen Taskforce sind, die – nach Anforderung durch die Bundesländer – unmittelbar in Ausbruchssituationen unterstützen kann.

### Infobox 3 Containment Scouts

- Im Auftrag des Robert Koch-Instituts hat das Bundesverwaltungsamt bereits im Frühjahr 2020 ca. 500 sogenannte Containment Scouts (CS) eingestellt, die die Gesundheitsämter vor Ort bei der Kontaktpersonennachverfolgung unterstützen.
- CS sind in der Regel Studierende der Medizin oder anderer Gesundheitswissenschaften, die – nach erfolgreicher Bewerbung – mit RKI-Materialien geschult werden (u. a. Einführung in die Infektionsepidemiologie und Ausbruchsuntersuchung, Umgang mit Meldesystem und Datenbanken).
- CS arbeiten vor Ort in den Gesundheitsämtern und helfen dabei, Kontaktpersonen nachzuverfolgen.
- Zusätzlich zu den lokalen Containment Scouts wurden 20 mobile Scouts eingestellt, die – bei Bedarf und unter Koordination des RKI – bundesweit überlastete Gesundheitsämter für jeweils 2–3 Wochen unterstützen können [17].

Ausbrüche von Infektionskrankheiten werden zuweilen als „Experimente der Natur“ bezeichnet. Diese können sich auch in bestimmten Settings ereignen, in denen sich wissenschaftliche Studien aus ethischen Gründen verbieten. So ermöglichen Ausbrüche Vergleiche zwischen verschiedenen Bevölkerungsgruppen, die anderweitig oft nicht genauer betrachtet werden können. Ausbruchsuntersuchungen können Rückschlüsse auf wichtige Kenngrößen eines noch weitgehend unbekannten Erregers erlauben. Darüber hinaus ermöglichen sie es, systematisch Erfahrungen zu sammeln, um eine Evidenzbasis aufzubauen, die für das Management von Ausbrüchen oder Einzelfällen benötigt wird, aber auch um präventive Maßnahmen bzw. Empfehlungen zu formulieren. Krankenhaus- oder pflegeheimassoziierte Ausbrüche spielen sich in einem speziellen Setting ab – die betroffenen Personen sind im Allgemeinen besonders vulnerabel und die beteiligten Berufsgruppen unterscheiden sich.

Ziel dieses Beitrags ist es, anhand von Beispielen für Ausbruchsuntersuchungen der RKI-Feldteams im Zeitraum Februar bis Oktober 2020 aufzuzeigen, inwieweit durch sie Erkenntnisse zu spezifischen epidemiologischen Fragestellungen gewonnen werden konnten, was die Teams zum Management der Ausbrüche beitragen konnten und welche präventiven Empfehlungen gegeben wurden. Dabei werden auch mehrere Erfahrungen aus Krankenhaus- bzw. Pflegeheimausbrüchen betrachtet.

## Einsätze mit Erkenntnissen zu Übertragung und Ausbreitung

### Erster Ausbruch in Deutschland, Bayern, Januar/Februar 2020

Der erste Ausbruch in Deutschland wurde bekannt, nachdem ein in Bayern ansässiger Autozulieferer von Kollegen aus China darauf hingewiesen wurde, dass eine chinesische Mitarbeiterin, die zwischenzeitlich in Deutschland tätig war, in Shanghai positiv auf SARS-CoV‑2 getestet worden war. Es stellte sich heraus, dass eine Vielzahl von Mitarbeitenden des Autozulieferers exponiert war. Mithilfe epidemiologischer und molekularbiologischer Methoden (Sequenzierung) rekonstruierte das Team die Infektionsketten [3]. Insgesamt infizierten sich im Zuge des Ausbruchs 16 weitere Personen mit dem Erreger.

Eine besonders relevante Erkenntnis aus dieser Ausbruchsuntersuchung war, dass Übertragungen auch ohne „typische“ Symptomatik, wie z. B. Fieber oder Husten, sowie auch präsymptomatisch erfolgen konnten. Das war zu diesem Zeitpunkt noch nicht bekannt. In mehreren Fällen war die Inkubationszeit sehr kurz. So wurden sehr früh wichtige Eigenschaften des Erregers deutlich, die vor allem nicht mit denen übereinstimmten, die vom SARS-CoV-1-Erreger von 2003 bekannt waren. Dessen im Krankheitsverlauf späte Infektiosität hatte die Eradikation durch klassische Public-Health-Maßnahmen erheblich begünstigt. In Deutschland wurde aufgrund dieser Erkenntnisse ab dem Auftreten der ersten SARS-CoV-2-Fälle empfohlen, Kontaktpersonen bereits ab dem zweiten Tag vor Erkrankungsbeginn des Primärfalles zu ermitteln.

### Ausbruch in einer Arztpraxis, März 2020

Ende Februar/Anfang März 2020 führte ein COVID-19-Ausbruch innerhalb einer Arztpraxis zu einem großen Infektionscluster in Bayern. Eine infizierte Ärztin und ein infizierter Arzt hatten vor Symptombeginn und Diagnose Patientinnen und Patienten behandelt. Der Kontakt mit diesen erfolgte dabei mit und ohne Mund-Nasen-Schutz. Es wurde eine retrospektive Kohortenstudie durchgeführt, in der das Infektionsrisiko der exponierten Patientinnen und Patienten untersucht wurde. Verglichen wurden dabei die Unterschiede in der Länge des Kontakts, der Art des Kontakts (Distanz, körperliche Untersuchung) und den jeweils verwendeten Infektionsschutzmaßnahmen (Mund-Nasen-Schutz) zwischen an COVID-19 Erkrankten und Nichterkrankten. Es zeigte sich, dass das Infektionsrisiko tendenziell erhöht war bei Kontakt mit einem der Ärzte, Kontakt in der präsymptomatischen Phase und Kontakt ≥ 10 Minuten. Verringert wurde das Risiko, wenn die Ärztin oder der Arzt einen Mund-Nasen-Schutz trug. Alle COVID-19-Fälle, die in diesem Cluster entdeckt wurden, hatten Kontakt mit einem der Ärzte, als diese oder dieser keinen Mund-Nasen-Schutz trug, und/oder hatten einen Kontakt, der ≥ 10 Minuten dauerte.

Die Ergebnisse sprechen für die Verwendung eines Mund-Nasen-Schutzes bei ärztlichem Personal und eine kurze Kontaktzeit (unter 10 Minuten), um das Übertragungsrisiko zu minimieren [4]. Die Ausbruchsuntersuchung unterstützt damit die aktuellen Richtlinien der WHO und des European Centre for Disease Prevention and Control (ECDC), nach denen alle Mitarbeitenden im Gesundheitswesen bei der Arbeit einen medizinischen Mund-Nasen-Schutz tragen sollten, insbesondere in Gebieten mit einem hohen Anteil nicht nachvollziehbarer Übertragungen [5–7].

### Ausbrüche in der Allgemeinbevölkerung in einem Landkreis in Bayern, März 2020

Ende Februar/Anfang März 2020 entwickelte sich ein COVID-19-Ausbruch mit insgesamt 59 Fällen in einem Landkreis in Bayern. Bei der Verbreitung hat vermutlich auch die Exposition bei Karnevalsveranstaltungen eine Rolle gespielt. Viele Fälle waren bei der Identifizierung (noch) asymptomatisch. Das Team befragte die infizierten Personen zu ihrer Symptomatik, ihrer Infektionsquelle und ihren Kontaktpersonen.

Es konnte keine laborbestätigte Übertragung von SARS-CoV‑2 ausgehend von asymptomatischen Fällen beobachtet werden. Das höchste Übertragungsrisiko wurde bei präsymptomatischen Fällen gesehen. Daher wird empfohlen, enge Kontaktpersonen von Infizierten so schnell wie möglich zu identifizieren und unter Quarantäne zu stellen. Aus den gesammelten Daten innerhalb des Clusters konnte ein mittleres serielles Intervall von 4,7 Tagen berechnet werden. Die mittlere Generationszeit lag bei 4,8 Tagen, die mittlere Inkubationszeit wurde auf 5 Tage geschätzt [8].

### Ausbrüche in Wohngebäuden, Juni 2020

2 Ausbruchsgeschehen in jeweils mehreren Gebäuden mit teilweise prekären Wohnbedingungen betrafen eine vergleichsweise junge Population mit einem Altersmedian zwischen 19 und 23 Jahren. Viele der Bewohnerinnen und Bewohner waren durch strukturelle Diskriminierung von Armut und sozialer Ausgrenzung betroffen. In den Wohngebäuden wohnten viele Familien mit kleinen Kindern in beengten Wohnverhältnissen.

Der Anteil an asymptomatischen Fällen war mit 45 % höher als in vielen anderen Settings (mit meist höheren Altersgruppen), was vermutlich in der hohen Zahl der durchgeführten Tests begründet liegt. In einem der beiden Ausbrüche, der 2 verschiedene Wohnhäuser betraf, waren die Anteile der Fälle in der Altersgruppe 0–17 Jahre (37 % bzw. 46 %) überproportional hoch im Vergleich zum Anteil bei der gesamten Wohnbevölkerung (13 % bzw. 33 %). Im anderen Ausbruch entsprach der Anteil der jungen Altersgruppe dem der Wohnbevölkerung. Die Gründe dafür sind unklar.

Auch bei dieser vergleichsweise jungen Bevölkerung kann es zu schweren Verläufen kommen, 2 unter 30-jährige Infizierte mussten intensivmedizinisch betreut werden. Eine Person (>60 Jahre) verstarb. Möglicherweise spiegelt sich hier auch das erhöhte Risiko für schwere Krankheitsverläufe beim Vorliegen von prädisponierenden Vorerkrankungen wider, deren Prävalenz in einer von Armut geprägten und von Diskriminierung betroffenen Bevölkerung erhöht ist. Bei einem der Ausbrüche befanden sich 48 % der SARS-CoV-2 Fälle in der Gruppe 0–18 Jahre im Vergleich zu 7 % und 26 % Fällen unter allen Getesteten in den verschiedenen Wohngebäuden. Diese untypische Verteilung wurde auf mögliche Übertragungen im Rahmen von Kontakten in den Haushöfen zurückgeführt.

### Ausbrüche unter Chormitgliedern, März 2020

Im März 2020 ereigneten sich Ausbrüche unter den Mitgliedern zweier Chöre mit sehr unterschiedlichen Erkrankungsraten von etwa 80–90 % bzw. 20 %. Die Umstände der Ausbrüche wurden untersucht hinsichtlich der wahrscheinlichen Quell- bzw. Primärfälle, deren Kontakte zu Personen im Umkreis von 1,5–2 m (Nahfeld), ihrer Aktivitäten (Dauer von Singen bzw. Sprechen) während der Proben, der Menge emittierter Partikel beim Singen bzw. Sprechen sowie der Raum- und Lüftungssituation zum Zeitpunkt der Exposition.

Hinsichtlich dieses Ausbruchs soll noch analysiert werden, inwiefern die geschilderten Faktoren die unterschiedlichen Anteile Erkrankter erklären können, ob und in welchem Ausmaß eine Nah- bzw. Fernfeldexposition, aber auch die Dauer einer solchen Exposition zu dem Übertragungsgeschehen beigetragen haben.

### Ausbrüche in Lebensmittel verarbeitenden Betrieben

In mehreren Fleisch verarbeitenden Betrieben kam es in der Belegschaft zu außergewöhnlich hohen Anteilen von Infizierten. Durch epidemiologische und molekularbiologische Untersuchungen in einer der Firmen sollen die Faktoren aufgedeckt werden, die diesen Ausbruch begünstigt haben.

Die Untersuchungsergebnisse zu diesem Ausbruch stehen noch aus. Es soll versucht werden, den separaten Beitrag verschiedener Faktoren, wie z. B. der Arbeitsbedingungen in der Firma (Nähe der Arbeitenden zueinander, Arbeitstempo bei z. T. körperlich schwerer Arbeit, Temperatur, Luftqualität) als auch der Bedingungen außerhalb der Firma (z. B. Wohnverhältnisse, Gruppentransporte), herauszuarbeiten.

### Ausbruch in der Allgemeinbevölkerung und in Altenheimen, März/April 2020

Der Landkreis Tirschenreuth im nördlichen Bayern meldete zu Beginn der Pandemie einen rapiden Anstieg der Fallzahlen, kombiniert mit einem hohen Fall-Verstorbenen-Anteil. Das Ausbruchsteam analysierte die Falldaten im Meldesystem und ergänzte qualitative und quantitative Informationen aus dem Gesundheitsamt. Daten zu durchgeführten Tests wurden von der bayerischen Landesbehörde und örtlichen Laboren eingeholt, Maßnahmen für die Allgemeinbevölkerung sowie Alten- und Pflegeheimbewohnende wurden überprüft. Die ersten 110 Fälle (nach Erkrankungsdatum) wurden zu bekannten Infektionsrisiken (Kontakt zu bestätigtem Fall, Rückreise aus Risikogebiet, Teilnahme an Großveranstaltungen etc.) befragt. Zur Auswertung wurden der zeitliche Verlauf der Fälle beschrieben und das Infektionsgeschehen auf Gemeindeebene betrachtet. Alter, Geschlecht und Vorerkrankungen der betroffenen Personen sowie der Anteil an Bewohnenden von Heimen im Landkreis wurden mit Daten für das übrige Deutschland verglichen.

Es zeigte sich, dass die Infizierten im Landkreis signifikant älter waren und deutlich öfter in Alten- und Pflegeheimen betreut wurden, wo es zu mehreren Ausbrüchen mit hohen Erkrankungs- und Sterberaten kam. Gleichzeitig deuten der hohe Anteil an symptomatischen Fällen, der hohe Anteil positiver Tests sowie der steile Anstieg der epidemischen Kurve nach Symptombeginn auf unentdeckte asymptomatische Fälle hin. Somit wurde der Fall-Verstorbenen-Anteil vermutlich überschätzt.

Initial führte eine Kombination mehrerer Risikosituationen vermutlich zum starken Anstieg der Fallzahlen. In mehreren Alten- und Pflegeheimen waren sowohl Bewohnende als auch Beschäftigte stark betroffen. Früh eingeführte Maßnahmen auf lokaler, Landes- und Bundesebene wie Informationskampagnen, Verbot von Großveranstaltungen, Ausgangsbeschränkungen und verstärkte Hygienemaßnahmen in Alten- und Pflegeheimen trugen entscheidend zur Kontrolle des Ausbruchs bis Ende April 2020 bei [9].

### Ausbruch in einem Nachtclub, März 2020

Ende Februar/Anfang März 2020 ereignete sich ein COVID-19-Ausbruch in einem Berliner Nachtclub. Die meisten der zugehörigen COVID-19-Fälle waren auf den Besuch einer dortigen Tanzveranstaltung am 29.02.2020 zurückzuführen, weitere dem Ausbruch zugeordnete Fälle wurden beim Besuch einer Veranstaltung am 05.03.2020 im gleichen Nachtclub exponiert. Das Team führte Fallbefragungen und SARS-CoV-2-Antikörperbestimmungen bei Mitarbeitenden des Nachtclubs durch und initiierte molekulargenetische Untersuchungen.

Insgesamt konnten 74 COVID-19-Fälle mit dem Ausbruch in Zusammenhang gebracht werden. 74,3 % der Fälle infizierten sich vermutlich direkt im Nachtclub, die Mehrzahl davon im Rahmen der ersten Veranstaltung. Über die Hälfte (56 %) der an den betroffenen Abenden anwesenden Mitarbeitenden des Nachtclubs infizierte sich [10].

## Einsätze mit Erkenntnissen zu Präventions- und Infektionsschutzmaßnahmen

### Ausbruch auf einem Kreuzfahrtschiff, Mai 2020

Am 28.04.2020 legte in Cuxhaven (Niedersachsen) ein Kreuzfahrtschiff mit einem COVID-19-Verdachtsfall an Bord an. Das Kreuzfahrtschiff hatte fast 3000 Personen an Bord, die zum Großteil Crewmitglieder von anderen Schiffen waren, die in ihre Heimatländer zurückreisen sollten; Touristen waren nicht an Bord. Das Gesundheitsamt Cuxhaven identifizierte insgesamt 9 SARS-CoV-2-Fälle. Zu den Maßnahmen an Bord zählten die Absonderung von bestätigten Fällen, symptomatischen Personen und engen Kontaktpersonen in unterschiedlichen Bereichen des Schiffes. Die Isolation erfolgte anfangs vorrangig an Bord, später an Land. Alle Personen wurden angehalten allgemeine Hygienemaßnahmen zu beachten, u. a. Mund-Nasen-Schutz zu tragen. Trotz einer hohen Anzahl an Kontaktpersonen traten keine weiteren Fälle auf. Möglicherweise waren die Fälle zum Zeitpunkt ihrer Entdeckung nicht mehr infektiös oder Maßnahmen an Bord haben weitere Übertragungen verhindert.

Das Team unterstützte vor Ort bei der Lageanalyse, einer Schiffsbegehung und der Formulierung von Empfehlungen für das weitere Vorgehen in Absprache mit dem niedersächsischen Landesgesundheitsamt. Es beriet bezüglich der Einrichtung eines Ad-hoc-Lagezentrums und half, relevante Dokumentenvorlagen auszuarbeiten (Standard Operation Procedures – SOPs; Lagebericht etc.). Als ein Großteil der Personen, wie initial geplant, in ihre Heimatländer zurückreiste, unterstützte das RKI die internationale Kommunikation mit den Zielländern.

### Ausbruch in einer Erstaufnahmeeinrichtung für Asylsuchende, April 2020

Als zentrale Herausforderungen bei einem Ausbruch in einer Erstaufnahmeeinrichtung für Asylsuchende wurden zum einen die Unterbringungsbedingungen und zum anderen Schwierigkeiten in der Kommunikation zwischen Behörden und Bewohnenden identifiziert. Teilweise wurden drastische Maßnahmen wie Massenquarantäne und polizeiliche Bewachung von Gebäuden ergriffen.

Auf Bitte eines Bundeslandes, unter Beratung von Expertinnen und Experten und unter Berücksichtigung der Belange der Gesundheits- und Innenressorts des Bundes und der Länder wurden „Empfehlungen für Gesundheitsämter zu Prävention und Management von COVID-19-Erkrankungen in Aufnahmeeinrichtungen und Gemeinschaftsunterkünften für Schutzsuchende“ [11] entwickelt. Sie sollen Gesundheitsämter und Betreibende von Erstaufnahmeeinrichtungen und Gemeinschaftsunterkünften dabei unterstützen, präventive Maßnahmen zur Vermeidung von Infektionen und Ausbrüchen sowie, im Fall eines Ausbruchs, Infektionsschutzmaßnahmen effektiv umzusetzen. Die Empfehlungen betreffen die Information und Identifikation von Risikopersonen, die rasche Isolation von Fällen sowie die Quarantäne von engen Kontaktpersonen. Die engen Wohnbedingungen, die unterschiedlichen gesprochenen Sprachen sowie die Lebenssituation der Bewohnenden müssen in den Empfehlungen berücksichtigt werden und die bundeslandspezifischen Regelungen (Mindestabstand, Hygieneregeln) umsetzbar sein. Wichtig sind dabei eine barrierefreie Kommunikation mit Bewohnerinnen und Bewohnern sowie Mitarbeiterinnen und Mitarbeitern inklusive der Nutzung von Sprachmittlung sowie die Ermöglichung des Abstandhaltens durch räumliche Entzerrung und die Unterbringung in kleinen Wohneinheiten.

### Ausbruch in einem Landkreis in Sachsen-Anhalt

In einem Landkreis in Sachsen-Anhalt wurde das Gesundheitsamt in der Strukturierung schon gewonnener Informationen unterstützt [12]. Die Auswertungen zeigten, dass es wichtig ist, durch sorgfältige Anamnese die Infektionsquelle zu identifizieren (Rückwärtsermittlung), auch um evtl. verborgene Cluster nicht zu übersehen. Die Infektionsquelle kann Ausgangspunkt für weitere Infektionsketten oder -ereignisse sein. Eine größere Zahl neu entdeckter Fälle, die keiner Fallperson oder keinem schon bekannten Cluster zugeordnet werden können, weist auf verborgene Infektionsketten hin.

Darüber hinaus zeigte sich auch die große Bedeutung einer sorgfältigen „Vorwärtsermittlung“ (Kontaktpersonennachverfolgung), um die Entstehung von neuen Clustern zu verhindern. Besondere Aufmerksamkeit sollte Kontaktpersonen geschenkt werden, die beruflich oder privat Kontakte zu besonders vulnerablen Gruppen haben, z. B. in Altenpflegeheimen arbeiten. Abriegelungen von Institutionen oder Orten sollten zügig wieder aufgehoben werden, wenn es dort keine erkennbare unkontrollierte Ausbreitung (mehr) gibt, d. h., wenn z. B. alle oder die allermeisten neuen Fälle bekannten Infektionsquellen zugeordnet werden können, von denen keine Übertragung mehr ausgehen kann.

Sowohl bei der Rückwärts- als auch bei der Vorwärtsermittlung ist es wichtig, Cluster zu erkennen, die bei ausbleibender Identifizierung eine Erregerausbreitung beschleunigen können. Der Anteil an Fällen ohne Infektionsquelle kann ein guter Indikator für nicht identifizierte Cluster sein, auf der anderen Seite sollte über die Aufhebung strikter Maßnahmen nachgedacht werden, wenn Infektionsquellen weitgehend zugeordnet werden können. Zum Schutz von Personen mit hohem Risiko für schwere Verläufe hat die Erkennung von exponierten Personen mit Kontakt zu vulnerablen Gruppen besondere Bedeutung.

### Ausbrüche in Wohngebäuden verschiedener Großstädte, Juni 2020

RKI-Teams unterstützten das Management von verschiedenen COVID-19-Ausbruchsgeschehen in Wohnkomplexen mit meist engen Wohnverhältnissen. Das Quarantänemanagement wurde in jeder Stadt unterschiedlich gehandhabt. Bei einigen Ausbrüchen ordneten die Gesundheitsämter die Quarantäne für ganze Häuserblöcke an, da von einer Ausbreitung im Gebäude ausgegangen wurde und Infektionsketten sich nicht mehr nachvollziehen ließen. Teilweise wurde die Quarantäne mit Zäunen, Sicherheitspersonal und Polizei durchgesetzt. Die Testung aller Bewohnenden wurde teils freiwillig, teils verpflichtend durchgeführt.

Eine Herausforderung war die Kommunikation zwischen den Mitarbeiterinnen und Mitarbeitern des Gesundheitsamts und den Bewohnerinnen und Bewohnern, die nur geringe oder keine Deutschkenntnisse hatten. Die Quarantäneanordnungen waren nicht für alle Betroffenen verständlich. Die Teams formulierten Empfehlungen zur weiteren Eindämmung des Ausbruchsgeschehens und zur Verbesserung der Kommunikation mit Sprachmittlung und partizipativen Ansätzen.

Da die Wohnhausquarantäne auch nichtexponierte Personen betrifft, sollte sie eher vermieden werden. Dabei sollten die unterschiedlichen Lebensrealitäten der Bewohnerinnen und Bewohner wie Armut, mangelnde soziale Anbindung, prekäre Beschäftigungsverhältnisse und enge Wohnverhältnisse berücksichtig werden. Es muss bedacht werden, dass eine Quarantäne einschneidende Folgen haben kann, wie etwa den Verlust der Arbeit oder das Verpassen von Schulunterricht.

Die Heterogenität und die spezifischen Bedarfe der Bewohnerinnen und Bewohner (z. B. medizinische und Versorgungsbedarfe, gesprochene Sprachen, Literalität) sollten bei der Planung und Durchführung von Maßnahmen zur Infektionskontrolle berücksichtigt werden. Diskriminierung und Rassismus sollten als entscheidende Determinanten für gesundheitliche Ungleichheiten anerkannt werden. Darüber hinaus sollten Möglichkeiten zur räumlichen Trennung geschaffen werden, ggf. innerhalb des Wohngebäudes oder in anderen geeigneten Gebäuden. Vorbeugend können Hygienekonzepte für Wohngebäude eingefordert werden. Schließlich können gesetzliche Regelungen wie ein Wohnungsschutzgesetz den Kommunen ermöglichen, überfüllte, heruntergekommene und damit ungesunde Wohnungen für unbewohnbar zu erklären.

### Ausbruch nach Hochzeitsfeiern, August 2020

Im Rahmen zweier Hochzeitsfeiern und nachfolgender Infektionen in Schulklassen meldete ein Gesundheitsamt, dass die Kontaktpersonennachverfolgung nicht mehr vollständig durchführbar war, und bat das RKI um Amtshilfe bei der Aufklärung und Kontrolle des Ausbruchsgeschehens.

Das Team diskutierte das bestehende Konzept für die Umsetzung von Maßnahmen der Absonderung im Setting Schule und identifizierte die Notwendigkeit der Etablierung einer zentralen Informations- und Kommunikationsstruktur im Gesundheitsamt.

### Quarantäne von Rückkehrern aus Wuhan, Februar 2020

Eine wissenschaftliche Mitarbeiterin des RKI (PAE-Fellow) unterstützte das Gesundheitsamt Germersheim im Rahmen ihrer Abordnung an das rheinland-pfälzische Landesuntersuchungsamt beim Management der Quarantäne von aus Wuhan rückkehrenden Deutschen und ihren Familien (Repatriierten) in einer Bundeswehrkaserne. Sie nutzte diese Gelegenheit, um mit Unterstützung von weiteren Mitarbeiterinnen und Mitarbeitern eine freiwillige Befragung der Betroffenen durchzuführen. Dabei wurde deren Wahrnehmung der Quarantäne und die qualitative Bewertung von Planung und Durchführung der Maßnahme erfasst. Zusätzlich wurden die Verhaltensweisen vor der Repatriierung erfragt.

Trotz eines zeitlich kurzen Vorlaufs bei der Planung wurde die Quarantäne von den Betroffenen insgesamt positiv wahrgenommen. Bei zukünftigen staatlich durchgeführten Quarantänemaßnahmen sollten die Unterschiede in der Wahrnehmung von Männern und Frauen bedacht werden [13].

## Einsätze in Gesundheits- und Pflegeeinrichtungen

### Ausbruch in einem Altenpflegeheim in Sachsen-Anhalt, März 2020

Ein Gesundheitsamt in Sachsen-Anhalt untersuchte, unterstützt durch ein RKI-Team, einen Ausbruch in einem Altenpflegeheim. Die Befragungen des Gesundheitsamtes ergaben, dass es zu einem Eintrag von SARS-CoV‑2 durch eine leicht erkrankte Pflegekraft mit präsymptomatischer Übertragung gekommen war, deren Partner zuvor mit einem Atemwegsinfekt aus dem Skiurlaub zurückgekehrt war. Diese und andere Beobachtungen stützen die generelle Empfehlung für Personal in Altenpflegeheimen, präventiv einen Mund-Nasen-Schutz zu benutzen.

Der Ausbruch wurde nach etwa 2 Wochen entdeckt. Eine umgehende Isolierung, Kohortierung von Bewohnenden, Zuordnung von Pflegepersonal und Aufteilung nach Infektionsstatus (infiziert/ansteckungsverdächtig/nicht infiziert) führte zu einer Unterbrechung der Infektionsketten. Es kam im Rahmen des Ausbruchsgeschehens zu SARS-CoV-2-Übertragungen auf Besucher, die das Virus danach in ihrem privaten Umfeld weiterverbreiteten [12].

### Ausbruch in einem Krankenhaus in Brandenburg

Ein RKI-Team beriet das Gesundheitsamt und ein großes Klinikum im Rahmen eines großen nosokomialen Ausbruchs im März und April 2020, bei dem SARS-CoV‑2 in mehr als 100 Fällen bei Patientinnen und Patienten und Personal nachgewiesen wurde. Trotz umfangreicher Maßnahmen bis hin zu einem Aufnahme- und Verlegungsstopp gestaltete sich die Unterbrechung der Infektionsketten als herausfordernd. Durch Verlegungen innerhalb der Klinik und durch infiziertes Personal war es zu einer Ausbreitung in verschiedene Bereiche der Klinik und darüber hinaus in weiterbehandelnde Einrichtungen gekommen.

Ein umfangreiches systematisches Screening von Patientinnen und Patienten sowie Personal ermöglichte im Verlauf eine Differenzierung von Infizierten und Nichtinfizierten. Die Infektionsschutzmaßnahmen in Bereichen für besonders gefährdete Risikogruppen (z. B. Onkologie) wurden noch weiter erhöht. Nach wiederholten Screenings konnte eine effektive Kohortierung mit folgenden Gruppen implementiert werden: (1) Patientinnen und Patienten mit SARS-CoV-2-Nachweis, (2) Kontakte von Fällen (ansteckungsverdächtig) und (3) Patientinnen und Patienten, die weder einen SARS-CoV-2-Nachweis noch Kontakt zu einem bestätigten Fall hatten.

Erkenntnisse aus dem praktischen Ausbruchsmanagement unterstützten bei der Erstellung von Empfehlungen, wie z. B. „Management von COVID-19-Ausbrüchen im Gesundheitswesen“ [14] und „Optionen zur getrennten Versorgung von COVID-19-Fällen, Verdachtsfällen und anderen Patienten im stationären Bereich“ [15]. Durch frühzeitiges systematisches SARS-CoV-2-Screening und Kohortierung von Fällen können Infektionsketten frühzeitig unterbrochen werden. Wenn sich ein Ausbruchsgeschehen aber in verschiedenen Bereichen einer Klinik ausbreitet, bedarf es einschneidenderer Maßnahmen zur Ausbruchskontrolle.

### Telefonische Unterstützung bei Ausbrüchen in Krankenhäusern

Die Amtshilfe des RKI wird in vielen Fällen auch telefonisch geleistet, wenn ein Einsatz vor Ort nicht notwendig oder möglich ist. Bei verschiedenen nosokomialen Ausbruchsgeschehen bestand ein enger fachlicher Austausch zwischen Gesundheitsämtern, Krankenhäusern und RKI. Hier soll dieses Vorgehen anhand von zwei Beispielen skizziert werden.

In einer Berliner Klinik war es zu vermehrten SARS-CoV-2-Nachweisen beim Personal gekommen. Es war zunächst unklar, ob zwischen diesen Infektionen ein Zusammenhang bestand oder nicht. Gemeinsam mit dem Gesundheitsamt wurde die Klinik bei der Planung eines Screenings unterstützt, wobei gezielt Personal und einzelne Stationen untersucht wurden. Weiterhin wurde das Personal durch Gesundheitsamt und Klinik befragt. Es zeigte sich, dass die Expositionen überwiegend außerhalb der Klinik lagen und von keinem zusammenhängenden Ausbruchsgeschehen auszugehen war. Im Rahmen dieser Kooperation zeigte sich ein gemeinsames Interesse an offenen Fragen zur Verbreitung von SARS-CoV‑2 im Gesundheitswesen, weshalb eine weiterführende gemeinsame Untersuchung geplant und durchgeführt wurde.

In einer Klinik in Sachsen kam es zu einem nosokomialen Ausbruch, mit zunächst weniger als 10 Fällen bei Personal und Patientinnen/Patienten. Die Ursache für die Ausbreitung von SARS-CoV‑2 war vermutlich ein Eintrag der Infektionen durch medizinisches Personal. Durch eine schnelle und besonnene Reaktion vor Ort konnte die Erregerverbreitung jedoch schnell eingedämmt werden: Ein systematisches Screening wurde direkt nach der Ausbruchsdetektion durchgeführt. Fälle, Kontakte und Nichtfälle wurden schnell und effektiv räumlich sowie personell voneinander getrennt. Durch einen kurzzeitigen Aufnahmestopp bestanden hierzu die nötigen räumlichen und personellen Ressourcen. Die umfangreichen Maßnahmen zur Ausbruchskontrolle bei SARS-CoV-2-Ausbrüchen im Krankenhaus stellen eine logistische und finanzielle Herausforderung für die Kliniken dar. Der öffentliche Gesundheitsdienst kann die Krankenhaushygiene dabei unterstützen, Maßnahmen zu priorisieren und umzusetzen.

## Fazit

Die beschriebenen Ausbruchsuntersuchungen haben dazu beigetragen, das Wissen zu SARS-CoV‑2 zu erweitern. Dadurch konnten RKI-Empfehlungen formuliert oder untermauert werden. Das Management komplexer Situationen konnte unterstützt werden. Bei Weitem sind jedoch noch nicht alle offenen Fragen zu dem neuen Erreger geklärt. Möglichkeiten zur weiteren Untersuchung sollten daher auch zukünftig genutzt werden, mit dem Ziel, wissenschaftliche Erkenntnisse in praktikable Empfehlungen umzusetzen. Auch bei retrospektiven Datenanalysen mit speziellen Fragestellungen hilft das RKI gerne.

Als eine der Maßnahmen zur Stärkung des öffentlichen Gesundheitsdienstes (ÖGD) wurde zum 01.09.2020 beim Robert Koch-Institut als zentraler Ansprechpartner eine „Kontaktstelle für den ÖGD“ eingerichtet, die u. a. die Amtshilfe des RKI und die Zusammenarbeit mit den zuständigen Landesbehörden koordiniert. Auch die gemeinsame Umsetzung des elektronischen Melde- und Informationssystems wird hier abgestimmt. Durch diese zusätzlichen Kapazitäten werden weitere Möglichkeiten für die Beratung der Gesundheitsämter und die praktische Einsatzunterstützung vor Ort durch Feldteams geschaffen.

Damit der ÖGD langfristig qualitativ hochwertig und vielseitig bei Ausbrüchen aller Erreger besser reagieren kann, soll darüber hinaus in Zusammenarbeit mit den Akademien für den öffentlichen Gesundheitsdienst auch das interdisziplinäre Trainingsangebot intensiviert werden. Einige zentrale Empfehlungen der Joint External Evaluation der WHO (JEE), die im Jahr 2019 im Rahmen der Internationalen Gesundheitsvorschriften (IGV) in Deutschland durchgeführt wurde, werden hierdurch ebenfalls adressiert (JEE-Bericht noch nicht publiziert).

Systematische infektionsepidemiologische Ausbruchsuntersuchungen können zum Verständnis von Erregerausbreitung in der Bevölkerung – in einzelnen Bevölkerungsgruppen und in speziellen Settings – sowie von Infektionsketten und Interventionen beitragen. Ziel ist es, mit dem Wissen aus diesen Untersuchungen vor Ort die Weiterverbreitung direkt zu unterbrechen und den Erkenntnisgewinn weiterhin zu nutzen, um allgemeine Infektionsschutzmaßnahmen kontinuierlich zu verbessern.
